# Activity-regulated E3 ubiquitin ligase TRIM47 modulates excitatory synapse development

**DOI:** 10.3389/fnmol.2022.943980

**Published:** 2022-09-21

**Authors:** Gourav Sharma, Sourav Banerjee

**Affiliations:** National Brain Research Centre, Gurgaon, India

**Keywords:** synapse, spine, E3 Ub ligase, neuronal activation, TRIM47

## Abstract

The Ubiquitin Proteasome System (UPS) has been shown to regulate neuronal development and synapse formation. Activity-dependent regulation of E3 ligase, a component of the UPS that targets specific proteins for proteasome-mediated degradation, is emerging as a pivotal player for the establishment of functional synapses. Here, we identified TRIM47 as a developmentally regulated E3 ligase that is expressed in rat hippocampus during the temporal window of synapse formation. We have demonstrated that the expression of TRIM47 is regulated by the glutamate-induced synaptic activity of hippocampal neurons in culture. In addition, the activity-dependent enhancement of TRIM47 expression is recapitulated following the object location test, a hippocampus-dependent spatial memory paradigm. We observed that this enhancement of TRIM47 expression requires NMDA receptor activation. The knockdown of TRIM47 leads to an enhancement of spine density without affecting dendritic complexity. Furthermore, we observed an increase in excitatory synapse development upon loss of TRIM47 function. Comprehensively, our study identified an activity-regulated E3 ligase that drives excitatory synapse formation in hippocampal neurons.

## Introduction

Information transfer from one neuron to another requires a highly specialized structure known as a synapse (Südhof, 2021). Synapses are formed in the developing as well as in the adult brain while performing cognitive functions (Cohen-Cory, 2002; Zito and Svoboda, 2002; Cornelia Koeberle et al., 2017; Nguyen et al., 2020). Although the initial phase of synapse formation occurs without neuronal activity, the maturation of these nascent synapses requires neuronal activity (Fertuck and Salpeter, 1976; Salpeter and Harris, 1983; Rao and Craig, 1997; Hall and Ghosh, 2008; Lin et al., 2008). Synapse formation is initiated as early as postnatal day 1 (P1) (Fiala et al., 1998). The density of synapses gradually increases from P7 to P15 and reaches a peak at P21 after which newly established synapses undergo maturation till P60 (Harris and Stevens, 1989; Harris and Jensen, 1992; Lohmann and Kessels, 2014; Zhu et al., 2018; Cizeron et al., 2020). Synapse development in dissociated neuronal culture follows a similar temporal pattern as detected in postnatal rodents (Fletcher et al., 1994; Papa et al., 1995; Boyer et al., 1998).

The development of functional synapses relies on a myriad of factors that operate gene expression both at the transcriptional and post-transcriptional level. The transcriptional regulation of synapse formation has been extensively studied. These studies identified the involvement of immediate early genes, such as NPAS4, in inhibitory synapse development (Lin et al., 2008). A screen for calcium-regulated transcription factors identified MEF2A/D as a regulator of excitatory synapse formation (Flavell et al., 2006; Polleux et al., 2007). A genome-wide RNA interference (RNAi) screen has identified transcriptionally regulated cell adhesion proteins, such as Cadherin 11 and 13, as regulators of functional synapse development (Paradis et al., 2007). More recently, post-transcriptional control of synapse formation has gained significant attention. Among various post-transcriptional regulators, protein ubiquitination and subsequent degradation by the Ubiquitin Proteasome System (UPS) has emerged as a key regulator. The UPS involves an enzymatic cascade to ubiquitinate a subset of proteins by E3 ubiquitin ligase and degrade the ubiquitinated proteins by the proteasome (Mabb and Ehlers, 2010). An initial unbiased genetic screen identified *Highwire*, an E3 ubiquitin ligase in *Drosophila* and its homolog *rpm-1* in *Caenorhabditis elegans*, to be involved in the regulation of synapse development (Wan et al., 2000; Nakata et al., 2005). The Ube3A, an E3 ubiquitin ligase mutated in Angelman Syndrome, has been shown to ubiquitinate Arc and trigger its degradation by proteasome upon induction of neuronal activity (Greer et al., 2010). The Arc degradation regulates the insertion of AMPA-type glutamatergic receptor subunit on the post-synaptic membrane that governs the development of excitatory synapses (Greer et al., 2010). Emerging studies have pointed out the non-degradative functions of E3 ubiquitin ligases. Neuralized1 (Neurl1) ubiquitinates cytoplasmic polyadenylation element binding protein 3 (CPEB3) and promotes its enzymatic activity for polyadenylation and translational regulation by transcripts containing binding sites for CPEB3 (Pavlopoulos et al., 2011). The E3 Ubiquitin ligase activity of Neurl1 is enhanced upon exposure to a spatial memory paradigm leading to the growth of new dendritic spines and synthesis of AMPA-type glutamatergic receptor subunits GluA1 and GluA2 (Pavlopoulos et al., 2011). Activity-dependent self-polyubiquitination of E3 ubiquitin ligase Rnf2 stabilizes its function and prevents its degradation *via* Ube3A-mediated polyubiquitination (Kumari et al., 2017). The self-polyubiquitination of Rnf2 regulates functional synapse development by modulating the insertion of AMPA-type glutamatergic receptors on the post-synaptic membrane (Kumari et al., 2017). Our screen for activity-regulated E3 ubiquitin ligase identified several E3 ubiquitin ligases, such as TRIM9, TRIM47, and PDZRN3 that could potentially regulate synapse formation (Kumari et al., 2017). Previous studies have implicated TRIM47, a RING domain containing E3 ubiquitin ligase, in the regulation of glioma cell proliferation and migration (Chen et al., 2020) and tumor progression in pancreatic cancer (Li et al., 2021). However, the function of TRIM47 remains elusive in the nervous system. These observations prompted us to evaluate the importance of TRIM47 in functional synapse development in hippocampal neurons.

In this study, we have identified TRIM47 as a synapse-associated E3 ubiquitin ligase and expressed during the temporal window of synapse formation. Our study demonstrated that TRIM47 is localized in neuronal dendrites and synapses of developing hippocampal neurons. We observed that glutamate-induced neuronal activity and exposure to a spatial learning paradigm enhance the NMDA-dependent expression of TRIM47. The knockdown of TRIM47 enhances spine density without affecting dendritic development. The loss of TRIM47 increases excitatory synapse formation. Our data establish TRIM47 as a negative regulator of the dendritic spine and synapse development.

## Materials and methods

### Primary hippocampal neuronal culture

Rat (Sprague-Dawley) primary hippocampal neuronal cultures were prepared and maintained as described previously (Kaech and Banker, 2006). Briefly, hippocampi were dissected out from embryonic day 18 (E18) pups, treated with trypsin (0.25%), and dissociated by trituration to a make single cell suspension. Cells were plated onto poly-L-lysine (1 mg/ml) coated glass coverslip (200–300 cells/mm^2^). About 190–200 cells/mm^2^ were used for imaging experiments and 300 cells/mm^2^ were used for all biochemical experiments. Neurons were maintained in the Neurobasal medium (Gibco, #21103-049) containing B27 supplements (Gibco, #17504-001) (NB27) at 5% CO_2_ and 37°C. Animals were generated at the animal house of the National Brain Research Centre and experiments were performed as per the guidelines approved by the Institutional Animal Ethics (IAEC) committee of the National Brain Research Centre.

### Lentivirus production and transduction

Lentivirus preparation and transduction into hippocampal neuronal cultures were performed as described previously (Banerjee et al., 2009). shRNA against TRIM47 was cloned into *Mlu*I and *Cla*I sites of the pLVTHM vector (Addgene plasmid #12247) and verified by sequencing. pLVTHM vectors containing TRIM47 shRNA (shRNA #1: ACTTGGTGAAGCTGAGTTCCC) or (shRNA #2: AGAAAGCTGACTGAATCCGCC) and non-targeting control (Universal Control: ATCTCGCTTGGGCGAGAGTAAG) were used for lentivirus preparation. *Escherichia coli* Stbl3 strain was used to propagate shRNA containing pLVTHM plasmid and DH5α was used to propagate psPAX2 (Addgene plasmid #12260) packaging plasmid and the pMD2.G (Addgene plasmid #12259) envelope plasmid. Purified plasmids were prepared by the Endotoxin-free Maxiprep kit (Qiagen). Lentiviruses were produced by co-transfecting 20 μg transfer vector (mCherry cassette under EF1α promoter and shRNA cassette against TRIM47 or non-targeting control under H1 promoter in pLVTHM plasmid), 15 μg psPAX2, and 6 μg pMD2.G into HEK293T cells. The cells were grown in low glucose DMEM media (Gibco) with 10% Fetal Bovine Serum (FBS) (Gibco) and maintained at 5% CO_2_ and 37°C. HEK293T (2 × 10^6^) cells were transfected by the calcium phosphate method. Following transfection, the media containing the transfection mixture was replaced with the fresh media after 8 h. Culture supernatant containing lentivirus particles were collected 72 h post-transfection and viruses were concentrated by ultracentrifugation. Viral titers were determined by infecting HEK293K cells followed by counting cells using a fluorescence-based cell counter. Titers were typically 1–3 × 10^7^ TU/ml.

RNAi was performed by infecting primary rat hippocampal neuronal cultures at *Days-In Vitro* (DIV) 3 with lentivirus expressing shRNAs against TRIM47 and non-targeting control. Virus infection was tracked by visualizing mCherry expression in infected neurons. The efficiency of knockdown was verified by preparing lysates from TRIM47 and non-targeting shRNA virus-infected neurons at DIV 21 in Laemmli buffer and resolving equal volumes on sodium dodecyl-sulfate polyacrylamide gel electrophoresis (SDS-PAGE). We have used two shRNAs against TRIM47 (Construct 1 and 2) for its effective knockdown and also a non-targeting shRNA to eliminate the possibility of an off-target effect. Viral infections were performed at a multiplicity of infection (MOI) of 2 for 6 h following which lentivirus-containing media was replaced with fresh neurobasal media supplemented with B27 (NB27). Neurons were incubated up to DIV 21 prior to immunocytochemistry and imaging experiments.

### Stimulation of hippocampal neurons

Hippocampal neurons (DIV 7) were stimulated by glutamate (20 μM) for 45 min as described previously (Banerjee et al., 2009) with minor modifications. Neurons were incubated in low-KCl-HBS (290 mOSm) [110 mM NaCl, 5.4 mM KCl, 1.8 mM CaCl_2_, 0.8 mM MgCl_2_, 10 mM D-glucose, and 10 mM HEPES-KOH (pH 7.4)] for 60 min prior to stimulation. To block NMDA receptor function, neurons were pretreated with its antagonist, AP5 (50 μM) for 50 min. To block Ca^2+^ activity, neurons were pretreated with EGTA (5 μM) for 50 min. Furthermore, neurons were stimulated with NMDA (20 μM), in low-KCl-HBS for 45 min. AP5 and EGTA remained in the incubation buffer for the entire duration of the experiment. After stimulation, neurons were harvested for protein lysate preparation using 1X Laemmli buffer.

### Western blot analysis

Samples from the stimulated or un-stimulated neurons or lentivirus infected neurons from 2 to 3 coverslips for each experimental condition were lysed using 1X Laemmli buffer and pooled together for western blot analysis. Equal volumes of lysates were resolved on 10% SDS polyacrylamide gel, transferred onto nitrocellulose membrane (Millipore), blocked with 5% skim milk (Merck), and probed with antibodies specific for TRIM47 (1:500; Bethyl Laboratories, #A301-194A), c-Fos (1:500; Calbiochem, #OP17), Tuj1 (1:10,000; Sigma, #T8578), and GAPDH (1:25000; Sigma, #G9545). For each lane, immunoblotting was performed with Tuj1 or GAPDH as the internal control to normalize protein levels.

To evaluate the developmental expression pattern of TRIM47, hippocampal tissue lysates from post-natal day pups (P0, P4, P7, P14, P21, P60, and P120) were analyzed by SDS-PAGE against TRIM47 antibody and Tuj1 expression was used for normalization.

For the spatial object recognition test-related protein expression analysis, the Long-Evans rats were euthanized and the dorsal hippocampus was isolated within 30 min after the test session for each animal to prepare a lysate using lysis buffer and 0.1% SDS. Protein estimation was done using Bradford’s protein estimation method Bradford (1976). An equal amount of protein was resolved on 10% SDS polyacrylamide gels and probed for TRIM47, c-Fos, and Tuj1.

Blots were detected by the chemiluminescence kit (Pierce) on X-ray films or the UVITEC gel documentation system. Band intensities were quantified using ImageJ (NIH). Statistical significance was measured by an unpaired two-tailed *t*-test or ANOVA.

### Synaptoneurosome preparation

Synaptoneurosomes were prepared as described previously (Steward et al., 1991). Briefly, hippocampi from P14 and P60 rat brains were homogenized in the homogenization buffer (0.32 M Sucrose, 2 mM HEPES pH 7.4, 0.1 mM EDTA, and 0.25 mM DTT) containing a protease inhibitor cocktail (Roche) using a Dounce tissue homogenizer (Kontes). Nuclei and cell debris were removed by centrifugation at 2,000 g for 2 min. The supernatant fraction containing the crude synaptoneurosome fraction was pelleted after centrifugation at 14,000 g for 10 min. The pellet (P2) was then resuspended in homogenization buffer (0.32M sucrose, 0.1 mM EDTA, 0.25 mM DTT, 200 mM HEPES-KOH, pH 7.4) and layered onto a 5–13% discontinuous Ficoll (Sigma) gradient that was equilibrated at 4°C for 60 min. The gradient was centrifuged at 20,000 g for 45 min at 4°C. A synaptoneurosome fraction located at the interface of 5–13% gradient was obtained. Ficoll was removed from the synaptoneurosome fraction by centrifugation at 20,000 g for 20 min at 4°C and resuspending the pellet in homogenization buffer. The synaptoneurosome pellet was resuspended in the synaptoneurosome buffer (10 mM Tris pH 7.5, 2.2 mM CaCl_2_, 0.5 mM Na_2_HPO_4_, 0.4 mM KH_2_PO_4_, 4 mM NaHCO_3_, and 80 mM NaCl) and lysed using 0.1% SDS. The protein concentration of total hippocampal lysate (mentioned here as crude, containing both nuclear and cytoplasmic fraction) and synaptoneurosome fraction was measured by the BCA protein estimation kit (Pierce). The quality of synaptoneurosome fraction was assessed using an equal amount of crude as well as synaptoneurosome lysate by western blot analysis using antibodies specific for the synaptic compartment (PSD95, 1:1,000; clone K28/43; Neuromab, #75-028 and Synapsin I, 1:5,000; Millipore, #AB1543), glia (GFAP, 1:1,000; abcam, #ab7260), and nucleus (Histone H1, 1:1,000; Millipore, #ab05-457).

### Calcium-phosphate transfection of neurons for spine analysis

Hippocampal neuronal cultures were transfected with a plasmid expressing enhanced green fluorescent protein (EGFP) under the chicken β-actin promoter (CAG-GFP) at DIV 2 as described previously (Jiang and Chen, 2006; Sun et al., 2013). Briefly, hippocampal neurons were transfected with 1 μg of CAG-GFP mixed with 25 μl of 2X-Hepes Buffer Saline (2x-HBS, pH 7.04) and 2.48 μl of 2.5M CaCl_2_ in ultrapure Milli-Q, bringing the volume up-to 50 μl per coverslip. Each mixing step was done with constant agitation, and the mixture was left undisturbed for about 15 min. About 50 μl of transfection mix was added to each well containing cultured neurons on glass coverslips and was mixed thoroughly. The cells were then incubated in 5% CO_2_ at 37°C for 1-h and 30 min. Each coverslip was then washed with Neurobasal media kept at 5% CO_2_ and 37°C at-least 3 times to wash off all the precipitates from the coverslip. The cells were then incubated in glial preconditioned NB27 at 5% CO_2_ and 37°C. Coverslips that showed green fluorescent protein (GFP) the next day (DIV 3) were then selected and transduced with lentivirus expressing shRNAs against TRIM47 and non-targeting control for 6 h and complete media was replaced. Half volume of glial conditioned NB27 media was replaced every third day for maintenance of the culture up to DIV 21.

### Immunocytochemistry for synapse formation assay

Hippocampal neurons (DIV 3) infected with lentivirus co-expressing mCherry along with non-targeting control shRNA, or shRNAs targeting TRIM47 were immunostained on (DIV 21) as described previously (Banerjee et al., 2009) with minor modifications. Neurons were washed three times in Minimum Essential Medium (MEM) with HEPES modification (MEM-H) (Sigma, #M2645-10L) and fixed with 4% paraformaldehyde (PFA) in 1X-PBS containing 4% sucrose at 37°C for 15 min. Following fixation, neurons were washed in MEM-H three times, permeabilized with 0.1% Triton-X-100 in 1X-PBS (PBST) for 90 s and washed one time in MEM-H. Neurons were incubated in blocking solution (1% bovine serum albumin [BSA] and 10% horse serum in MEM-H) at RT for 1 h. These neurons were incubated with antibodies specific for PSD95 (1:300; clone K28/43; Neuromab, #75-028.) and Synapsin I (1:1,000, Millipore, #AB1543) overnight at 4°C. Following primary antibody incubation, neurons were washed three times in MEM-H and incubated with respective secondary antibodies (mouse or rabbit) conjugated with Ms-Alexa–488 (Invitrogen Molecular probes, #A21202, for PSD95) or Rb-Alexa–633 (Invitrogen Molecular probes, #A21071, for Synapsin I) dyes at RT for 35 min. Neurons were washed three times in MEM-H, treated with methanol at –20°C for 5 min, air dried, and mounted on glass slides using Vectashield mounting medium containing DAPI (Vector Laboratory, #H1200).

### Immunocytochemistry for TRIM47 localization in hippocampal neurons

Hippocampal neurons (DIV 7 and DIV 21) were immunostained as described previously (Banerjee et al., 2009) with minor modifications. Neurons were washed three times in MEM with HEPES modification (MEM-H) (Sigma, #M2645-10L) and fixed with 4% PFA in 1X-PBS containing 4% sucrose at 37°C for 15 min. Following fixation, neurons were washed in MEM-H three times, permeabilized with 0.1% Triton-X-100 in 1X-PBS (PBST) for 90 s, and washed one time in MEM-H. Neurons were incubated in a blocking solution (1% BSA and 10% horse serum in MEM-H) at RT for 2 h. These neurons were incubated with antibodies specific for PSD95 (1:300; abcam, #ab12093) and TRIM47 (1:300, Bethyl lab, #A301-194A) overnight at 4°C. Following primary antibody incubation, neurons were washed three times in MEM-H and incubated with respective secondary antibodies (mouse or rabbit) conjugated with Rb-Alexa–488 (Invitrogen Molecular probes, #A11034, for TRIM47) or Goat-Alexa–594 (Invitrogen Molecular probes, #A11058, for PSD95) dyes at RT for 35 min. Neurons were washed two times in MEM-H, followed by 1 h blocking in the blocking solution and then incubated with Tuj1 antibody (1:10,000; Sigma, #T8578). Neurons were washed with MEM-H two times and then incubated with secondary antibody Ms-Alexa-647 (Invitrogen Molecular probes, #A21236, for Tuj1) followed by three MEM-H washes for 5 min each. Neurons were air dried and mounted on glass slides using Vectashield mounting medium containing DAPI (Vector Laboratory, #H1200).

### Immunohistochemistry

Long-Evans rats aged P14 and P60 were sedated using a lethal dose of Ketamine (300 mg/kg) and a Xylazine (30 mg/kg) cocktail. Rats were perfused intracardially with phosphate buffer saline (PBS—137 mM NaCl, 2.7 mM KCl, 8 mM Na_2_HPO_4_, and 2 mM KH_2_PO_4_) and 4% PFA in PBS. Brains were isolated and kept in 4% PFA in PBS for 2 days post perfusion. Brains were transferred to 30% sucrose solution in PBS till they settle down at the bottom and are ready for sectioning. About 50 μm sections were prepared using cryotome and mounted on gelatin-coated slides. Sections were washed with PBS three times for 5 min each and then permeabilized using 0.1% TritonX-100 in PBS (0.1% PBST) for 60 min. Sections were again washed with 0.1% PBST three times for 5 min each. Blocking was done using 2% BSA + 5% Horse Serum in 0.1% PBST at RT for 2 h. Primary antibody incubation for MAP2 (5 μg/ml, abcam, #ab28032) and TRIM47 (1:250, bethyl lab, #A301-194A) in blocking solution was done for 20 h at 4°C. Following primary antibody incubation, sections were washed three times in 0.1% PBST and incubated with respective secondary antibodies (mouse or rabbit) conjugated with Rb-Alexa–488 (Invitrogen Molecular probes, #A11034, for TRIM47) or Ms-Alexa–647 (Invitrogen Molecular probes, #A21236, for MAP2) dyes at RT for 4 h. Neurons were washed three times in 0.1% PBST and mounted using Vectashield (#H1200) mounting medium containing DAPI.

### Confocal microscopy and image analysis

Using a Nikon Ti-2 point scan confocal microscope with a Plan Apo λ 60X Oil NA, oil immersion objective at 1,024 × 1,024 pixel resolution and at ×0.7 optical zoom, 16-bit images of pyramidal neurons were acquired. High-magnification images were obtained using a Plan Apo 100× OIL DIC N2 oil immersion objective at ×2 optical zoom at 1,024 × 1,024 pixel resolution. We obtained 6–8 optical sections with 0.75 μm step size (×60), and a maximum intensity projection was created for image analysis. Furthermore, 8–16 optical sections with a 0.5 μm step size (×100) were deconvolved with Nikon elements AR software for spine analysis and synapse formation assay.

Alexa 488 (PSD95), mCherry (Lentivirus RNAi), and Alexa 633(Synapsin I) were visualized by excitation of the 488-nm argon ion laser, 561 nm Helium–Neon laser (HeNe1), and 640-nm Helium–Neon (HeNe2) laser, respectively. We have used emission filters BP 505–530 for GFP, LP 560 for Alexa 546, and the Meta setting for Alexa 633. All images were acquired with identical settings for laser power, detector gain, amplifier offset, and pinhole diameter for each experiment and between experiments. Image analysis was performed as described previously (Paradis et al., 2007) with minor modifications.

For analyzing PSD95 and Synapsin I colocalization puncta density values, maximum intensity projections were created from the optical sections obtained from the images of all three channels (488, 546, and 633). mCherry images of multiple frames of all the neurons were used to trace the dendrites using NeuronStudio software (CNIC, Mt Sinai School of Medicine). Synapse density was quantified as the overlap of images of all three channels (488, 546, and 633) using a custom program in Matlab software (Mathworks). For TRIM47 colocalization with PSD95 on Tuj1 labeled neurons, the channels were 488, 594, and 647, respectively, and localization was quantified as the overlap of signals in all three channels.

The threshold for each channel (488/633 or 488/594) in each image was calculated as the mean pixel intensity for the entire image plus two standard deviations (SDs) above the mean. The threshold for GFP (488) was set at the mean pixel intensity plus one standard deviation for each image. A binary mask that included all pixels above threshold was created for each channel. Then, using the mask for each channel and trace files from NeuronStudio, regions of triple co-localizations lesser than 10 μm in size were considered as synaptic puncta. To calculate synapse density (per μm dendrite), the number of objects was divided by the length of the dendrite as measured using NeuronStudio traces. The synapse density for each condition within an experiment was calculated by averaging the synapse density from all of the images corresponding to a particular experimental condition. Statistical significance was assessed using a Student *t*-test and one-way ANOVA as indicated. All analyses were performed blind to the experimental condition.

For spine morphology analysis, neurons that showed expression of both mCherry (RNAi) and GFP (CAG-GFP transfection) were included. Neurons were processed using Neurolucida 360 (MBF Bioscience) to generate a three dimensional (3D) dendritic skeleton automatically using the Rayburst Crawl tracing algorithm. Following successful tracing of the dendritic branch, the spines were detected and classified automatically using the detect spine program feature. Structures that were not spines and spines that were ambiguous in shape were manually removed or reclassified. The statistical significance in spine morphology and density changes were assessed using a Student *t*-test and one-way ANOVA as indicated. All analyses were performed blind to the experimental condition.

### Sholl analysis

Sholl analysis was performed on neurons transduced with CAG-GFP and infected with lentivirus to evaluate the neuronal morphology post RNAi. SynD (Schmitz et al., 2011) was used for the analysis. The cell soma was determined and the intersections were counted in concentric circles with increasing radii of 10 μm from the center of the cell soma. A number of intersections were plotted against their distances from the center of the cell soma. The statistical significance between non-targeting control RNAi and TRIM47 RNAi was measured using the Kolmogorov–Smirnov (K–S) test.

### Spatial object recognition

The object recognition test performed is an adaptation of the traditional object recognition test for location memory in rats previously described (Cavoy and Delacour, 1993; Sutcliffe et al., 2007; Wimmer et al., 2012). The spatial object recognition test consists of 7 days of animal handling phase followed by 5 test sessions. In the first session, the animal is presented to the empty test arena (white floor and gray wall box with 80 cm × 80 cm × 50 cm dimensions along with spatial cues on its walls) for 10 min to be familiarized with the test environment, which differs from the home cage. Following the habituation session, the animal is returned to the home cage for a minimal time of not more than 5 min, which is necessary to clean the arena with 70% alcohol and to place the objects into the arena. In the next 3 phases that are spaced 5 min apart, the animal is allowed to explore two objects which remain at the same position in the box for 10 min in all three sessions. After the 4th session, the animal is returned to the home cage for 24 h following which the 5th session (test session) is carried out. In the test session, objects are either placed at the same location as in the last training session (object not displaced) or one of the two objects is moved to a different location (object displaced) with respect to the other object and the spatial cues on the wall of the arena, and the animal is allowed to explore the two objects freely for 10 min. The videos of the exploration sessions were recorded using an overhead mounted camera (Logitech HD webcam C270). The time spent exploring each object (interaction time) was recorded using MATLAB-based autotyping software (Patel et al., 2014) and manually by an observer blind to the experimental details and the identity of control and test animals. Exploration is defined as approaching the object with the nose closer than 2 cm. The recognition memory readout is the percentage of time spent with each object during test sessions (Denninger et al., 2018). Long-Evans rats of age 6–8 weeks were tested for object location memory (spatial object recognition test). Animals were generated at the animal house of the National Brain Research Centre and experiments were performed as per the guidelines approved by the IAEC of the National Brain Research Centre.

### Statistical methods

Statistical analyses were performed for all experiments. Data are represented as the percentage mean ± standard error of mean (SEM). The “*n*” number mentioned in each experiment refers to the number of independent experiments performed or for imaging experiments “*n*” is the number of neurons analyzed. Western blots were analyzed using an unpaired 2-tailed Student’s *t*-test assuming unequal variances or one-way ANOVA or as indicated. Synaptic puncta density analyses for co-localization of GFP/PSD95/Synapsin I puncta, spine density, and morphology analysis was performed using one-way ANOVA with Dunnett’s T3 multiple comparison test. We used the K–S test to determine the statistical differences in the distribution data from our Sholl analysis. The statistical significance was judged to be *p* < 0.05, and *p*-values are indicated in the respective figure legends. A statistical analysis was performed using GraphPad Prism 9.

## Results

### Activity-regulated TRIM47 expression during synapse development

**FIGURE 1:**
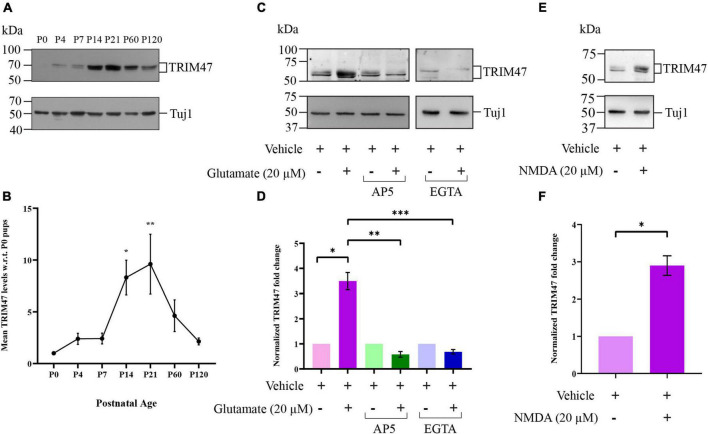
Developmental and activity-dependent expression of TRIM47. **(A)** A photomicrograph showing the postnatal developmental expression pattern of TRIM47 protein in rat hippocampus. **(B)** Quantification of western blot results is shown. *n* = 5, ^**^p < 0.0048, and *p < 0.0215. One-way ANOVA with Tukey’s test is conducted. **(C)** A photomicrograph showing western blot analysis of activity-induced TRIM47 protein expression following glutamate (20 μM) stimulation in the presence or absence of NMDA antagonist AP5 (50 μM) and chelating agent EGTA (5 μM). **(D)** Quantitation of TRIM47 expression is shown. *n* = 8; *p < 0.0002, ^**^p < 0.003, and ^***^p < 0.01; and an unpaired *t*-test with Welch’s correction. **(E)** A photomicrograph showing the TRIM47 level following NMDA (20 μM) stimulation. **(F)** Quantitation of TRIM47 expression after NMDA stimulation; *n* = 12; and ^****^p < 0.0001. An unpaired *t*-test with Welch’s correction is shown. The data represent the mean ± SEM.

To visualize the expression pattern of TRIM47 during the synapse formation, we measured the protein expression level at various stages of developing and adult hippocampus, starting from P0 (postnatal day 0) to P120. Our western blot analysis showed a detectable level of TRIM47 from P4 and its expression reaches to the maximum level at P21 (Figures 1A,B). We observed a significant increase in the level of TRIM47 at both P14 (8.30814 ± 1.671-fold increase, *p* < 0.0048) and P21 (9.60853 ± 2.887-fold increase, *p* < 0.0125) as compared with its level at P0 (Figures 1A,B). This observation indicates that the expression of TRIM47 coincides with the temporal window of synapse formation (Harris and Stevens, 1989; Harris and Jensen, 1992; Fletcher et al., 1994; Papa et al., 1995; Boyer et al., 1998; Fiala et al., 1998; Li and Sheng, 2003).

Synapse development has been shown to be regulated by neuronal activity (Nelson et al., 1990; Shatz and Katz, 1996; Zito and Svoboda, 2002; Yu et al., 2004; Andreae and Burrone, 2014). Synaptic activation triggers the translocation of proteasome machineries to dendritic spines and promotes protein ubiquitination *via* NMDA receptor activation (Bingol and Schuman, 2006). Prompted by these observations, we have analyzed the impact of neuronal activity on TRIM47 expression. Hippocampal neurons (DIV 7) were treated with glutamate (20 μM) for 45 min and the TRIM47 protein level was analyzed by western blot. We observed that the glutamate stimulation enhances the TRIM47 protein level (3.496 ± 0.3417-fold increase as compared with vehicle treated neurons, *p* < 0.0002, *n* = 8) (Figures 1C,D). To evaluate the contribution of NMDA receptor in the activity-regulated enhancement of TRIM47, hippocampal neurons (DIV 7) were treated with AP5 (50 μM), NMDA antagonist, in the presence or absence of glutamate. We observed that blockade of NMDA activity prevented activity-induced enhancement of TRIM47 (Figures 1C,D). However, AP5 alone did not affect the TRIM47 level (Figures 1C,D). Furthermore, we have tested whether NMDA receptor activation alone is sufficient for an enhanced expression of TRIM47. Our western blot analysis from NMDA-treated hippocampal neurons (DIV 7) demonstrated that the NMDA receptor activation alone is sufficient for increase in TRIM47 protein level (2.89 ± 0.91-fold increase as compared with vehicle treated neurons, *p* < 0.0001, *n* = 12) (Figures 1E,F).

The NMDA-induced Ca^++^ activity triggers protein ubiquitination (Bingol and Schuman, 2006; Djakovic et al., 2009; Xu et al., 2018). To evaluate the role of Ca^++^ influx in activity-dependent enhancement of TRIM47, neurons were stimulated with glutamate in the presence or absence of the chelating agent EGTA. Our western blot analysis demonstrated that chelation of Ca^++^ by the application of EGTA (5 μM) prevented glutamate-induced enhancement of TRIM47 (Figures 1C,D). However, EGTA alone did not influence the TRIM47 level (Figures 1C,D), indicating that NMDA-induced Ca^++^ activity influences enhancement of TRIM47.

**FIGURE 2:**
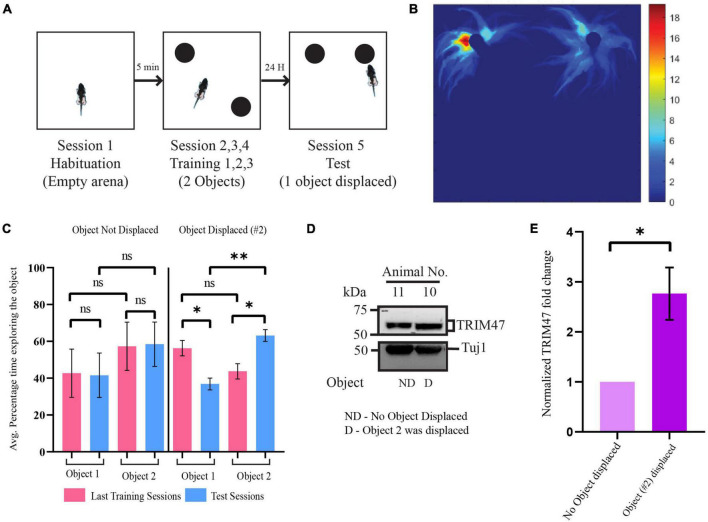
Enhancement of TRIM47 expression during spatial memory formation. **(A)** Schematic of the Spatial Object Recognition (SOR) memory test. **(B)** A representative heatmap showing the time spent by rat in the arena during the test session. **(C)** Quantitation of the average percentage time spent by rats exploring each object during the last training sessions and test sessions. Rats which were tested for object location memory where object location was same during the training and the test sessions (object not displaced or ND) and object location altered (object displaced or D) during test sessions. *p < 0.0114, ^**^p < 0.0012; *n* = 4. Data are shown as the mean ± SEM, one-way ANOVA, and Fisher’s least significant difference (LSD). **(D)** Enhancement of TRIM47 in the hippocampus was detected from animals showing preferential exploration of the displaced object. A photomicrograph showing TRIM47 and Tuj1 proteins expression levels in tissue isolated from dorsal hippocampi of rats subjected to a spatial object recognition memory test. ND—rats subjected to SOR where the object location was same during the test and training sessions; D—rats subjected to SOR where the location of one of the objects was changed during the test session relative to the training sessions. **(E)** Quantitation of western blot analysis post SOR. *p < 0.0434; *n* = 4. An unpaired *t*-test with Welch’s correction is conducted. Data represent the mean ± SEM.

Since TRIM47 expression is enhanced in hippocampal neuronal culture following the application of glutamate, we have tested whether neuronal activity could regulate TRIM47 expression *in vivo*. We have analyzed its abundance in the dorsal hippocampus during spatial memory formation. We have used object location task, a spatial memory paradigm that induces synaptic activity in the physiological environment (Oliveira et al., 2010; Barbosa et al., 2013; Cohen et al., 2013; Cinalli et al., 2020). Consistent with previous observations (Ennaceur et al., 1997; Ennaceur, 2010; Oliveira et al., 2010), we observed that rat showed preferential exploration of displaced object after the training in arena with two identical objects. The dorsal hippocampus from these rats were isolated and the TRIM47 level was analyzed by western blot. Our data showed an enhancement of TRIM47 (2.76 ± 0.52-fold increase, *p* < 0.0434, *n* = 4) in the dorsal hippocampus of the rats that preferentially explored the displaced object (Figures 2C–E).

### Localization of TRIM47 at the synapse

**FIGURE 3:**
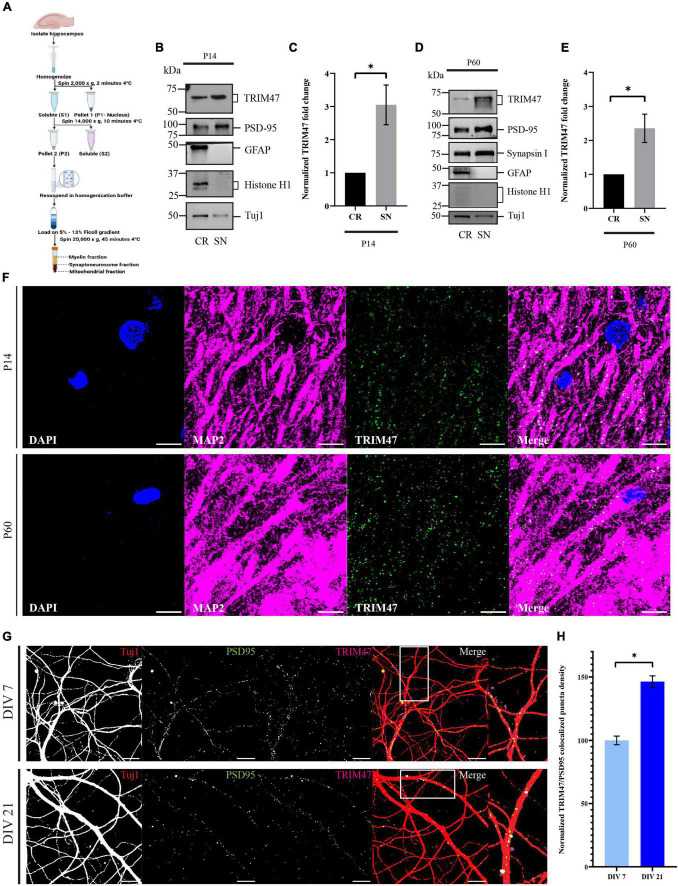
Localization of TRIM47 at the hippocampal synapse. **(A)** The schematic representation of synaptoneurosome preparation. Synaptoneurosome is prepared from rat hippocampus (P14 and P60) by ficoll density gradient (5–13%) centrifugation. **(B,D)** Synaptoneurosome purity is characterized by western blot analysis using antibodies specific for proteins enriched at the synapse (PSD95 and Synapsin I), astrocyte (GFAP), and nucleus (Histone H1). Tuj1 is used for normalization. Crude (CR) represents both nuclear and cytoplasmic proteins and synaptoneurosome (SN) represents synaptic proteins. Western blot showing synaptic localization of TRIM47. **(C,E)** Quantitation of TRIM47 protein levels showing synaptic enrichment. Data represent the mean ± SEM; *n* = 4; *p < 0.05; and an unpaired *t*-test with Welch’s correction. Icons in schematic representation **(A)** created in biorender.com. **(F)** Rat brain sections at P14 and P60 are immunostained for TRIM47, dendrites marked by MAP2, and sections are mounted in a medium containing DAPI. Scale—10 μm. **(G)** Neurons at DIV7 and DIV21 are immunostained for TRIM47 (magenta) as well as postsynaptic density protein PSD95 (green). Localization of TRIM47 at the synapses is visualized by apposing TRIM47 and PSD95 puncta onto hippocampal neurons labeled for Tuj1 (red). TRIM47/PSD95 co-localized puncta per micrometer of dendrite is measured to detect the puncta density. Scale: 10 μm. **(H)** Quantitation of synapse localized TRIM47 puncta density is measured from neurons at DIV7 and DIV21. *n* = 12–13; *p < 0.0001; and an unpaired *t*-test with Welch’s correction. Data represents the mean ± SEM.

Since TRIM47 showed an activity-dependent enhancement during the temporal window of synapse formation, we anticipate that the E3 ubiquitin ligase could possibly be localized at the synapto-dendritic compartment to drive synapse formation with spatial resolution. We have purified synaptoneurosome, a biochemical fraction enriched with synaptic compartment, from P14 and P60 rat hippocampi using ficoll density-gradient fractionation (Steward et al., 1991; Figure 3A. The purity of the synaptoneurosomal fractions were evaluated by immunoblotting of synaptic proteins, such as PSD-95 and Synapsin I, which showed enrichment in the synaptoneurosomal fraction as compared with the crude fraction. The glial protein GFAP and nuclear protein histone H1 being absent from the synaptoneurosomal fractions further confirmed their purity (Figures 3B,D). We detected that endogenous TRIM47 was enriched (3.05 ± 0.5992-fold increase, *p* < 0.0417, *n* = 4) and at P60 (2.35 ± 0.4172-fold increase, *p* < 0.0476, *n* = 4) in the synaptic fraction compared with the total cellular (crude) fraction (Figures 3C,E). We immunostained P14 and P60 rat brain sections using TRIM47 and the dendritic marker microtubule-associated protein 2 (MAP2). TRIM47 was found in a variety of brain areas, including the hippocampus (Supplementary Figure 1). Our data detected TRIM47 puncta in dendrites of hippocampal neurons from rat brain at P14 and P60 (Figure 3F). Furthermore, synaptic localization in dissociated hippocampal neuron (DIV 7 and DIV 21) were detected by the colocalization of TRIM47 puncta with PSD95 labeled synaptic puncta (Figure 3G). We observed that the TRIM47 showed an increased colocalization with PSD95 at DIV 21 (46.3 ± 4.52% increase, *p* < 0.0001, *n* = 12–13) as compared with neurons at DIV 7 (Figure 3H).

### Role of TRIM47 in dendritic development

**FIGURE 4:**
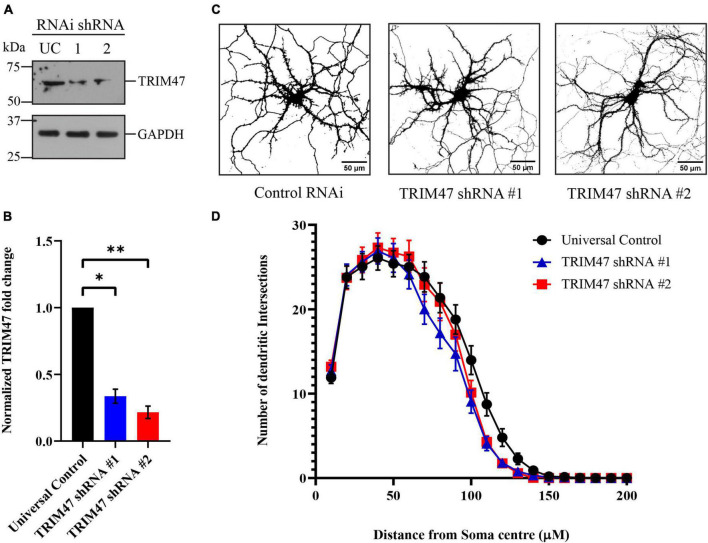
Effect of TRIM47 knockdown in dendritic development. **(A)** Photomicrograph showing effective knockdown of TRIM47 protein level in hippocampal neurons (DIV 21) by two distinct shRNAs. **(B)** Quantitation of TRIM47 normalized protein level following knockdown. *n* = 3; *p < 0.006 and ^**^p < 0.003; and an unpaired *t*-test with Welch’s correction. **(C)** Representative photomicrographs showing dendritic complexity (DIV 21) from hippocampal neurons transduced with lentivirus expressing two distinct shRNAs against TRIM47 (DIV 3). Dendritic development is analyzed by Sholl analysis. Scale as indicated. **(D)** Quantitation of Sholl analysis showing dendritic intersections at concentric circles drawn at 10 μm radius interval from the center of the soma. *n* = 30–32. One-way ANOVA with Dunnett’s T3 multiple comparisons test was used to measure the statistical significance. *P* < 0.92 (control RNAi *vs.* TRIM47 knockdown by shRNA#1) and *p* < 0.98 (control RNAi *vs.* TRIM47 knockdown by shRNA#2); and the Kolmogorov–Smirnov test. Data represents mean ± SEM.

Enhanced expressions of TRIM47 starting from P4 to P21 coincides with the temporal window of dendritic development (Pokorný and Yamamoto, 1981; Kroon et al., 2019). We have analyzed the importance of TRIM47 in dendritic development following the effective knockdown of TRIM47 by two distinct shRNAs. Hippocampal neurons were transduced (DIV 3) with lentivirus expressing shRNAs against TRIM47. The efficacy of knockdown in hippocampal neurons (DIV 21) was analyzed by western blot. We observed that both shRNA against TRIM47 led to significant reduction of the protein (shRNA #1—66.4 ± 5.28% decrease, *p* < 0.0063, *n* = 3; and shRNA #2—78.43 ± 4.59% decrease, *p* < 0.0034, *n* = 3) (Figures 4A,B). To evaluate the importance of TRIM47 in dendritic development, hippocampal neurons (DIV 2) were transfected with the chicken β-actin promoter driven EGFP (CAG-GFP) to visualize neuronal processes, and these neurons (DIV 3) were transduced with lentivirus expressing two distinct shRNAs against TRIM47 along with mCherry. Sholl analysis was performed using confocal images from neurons (DIV 21) expressing non-targeting control shRNA or shRNAs against TRIM47. We observed that knockdown of TRIM47 by two distinct shRNAs has no effect on dendritic development (Figures 4C,D).

### TRIM47 affects dendritic spine density and spine morphology

**FIGURE 5:**
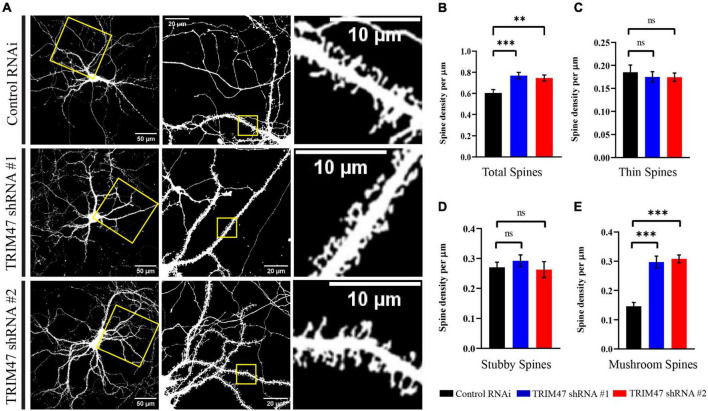
Dendritic spine morphology following TRIM47 knockdown. **(A)** Representative photomicrograph showing dendritic spine morphology from hippocampal neurons (DIV 21) that are transduced with two shRNAs against TRIM47 (DIV 3) and transfected with chicken βactin promoter driven GFP to visualize dendritic spine. Scale as indicated. **(B)** Quantitation of the total dendritic spine density following TRIM47 knockdown. Spine density per micrometer of dendritic length is measured from confocal images using Neurolucida 360. ^***^p < 0.0009 for shRNA#1, ^**^p < 0.0029 for shRNA#2. **(C–E)** Quantitation of thin spine **(C)**, stubby spines **(D)**, and mushroom spines **(E)** following TRIM47 knockdown. ^***^p < 0.0001 for **(E)**, ns = not significant for **(C,D)**. *n* = 16–25. One-way ANOVA with Dunnett’s T3 multiple comparisons test. Data are shown as the mean ± SEM.

Factors influencing dendritic spine structure have been shown to influence development and functions of synapse (Sala et al., 2001; Pavlopoulos et al., 2011; Gao et al., 2017; Sell et al., 2021). We have tested the impact of TRIM47 knockdown on dendritic spine development. Hippocampal neurons (DIV 3) were transduced with lentivirus expressing shRNAs against TRIM47 along with mCherry. Prior to transduction, CAG-GFP was transfected in hippocampal neurons (DIV 2) to visualize the dendritic spine. These EGFP-labeled dendritic spines were imaged from hippocampal neurons (DIV 21) to evaluate their structural change following the loss of TRIM47 function. We observed that knockdown of TRIM47 led to a significant increase (16.34 ± 3.2%, *p* < 0.0009 for shRNA#1; and 14.11 ± 2.85%, *p* < 0.0029 for shRNA#2) of total dendritic spine density (Figures 5A,B). We have measured spine type and observed that the loss of TRIM47 enhances mushroom spine (15.09 ± 2.05%, *p* < 0.0001 for shRNA#1; and 16.18 ± 1.36%, *p* < 0.0001 for shRNA#2) without influencing thin (shRNA #1—1.03 ± 1.92%, *p* < 0.8; and shRNA #2—1.09 ± 1.8%, *p* < 0.75) and stubby spines (shRNA #1—2.18 ± 2.57%, *p* < 0.59; and shRNA #2—0.7 ± 3.16%, *p* < 0.95) (Figures 5A–E). Our observations indicate that TRIM47 is a negative regulator of dendritic spine structure.

### TRIM47 regulates glutamatergic synapse density

**FIGURE 6:**
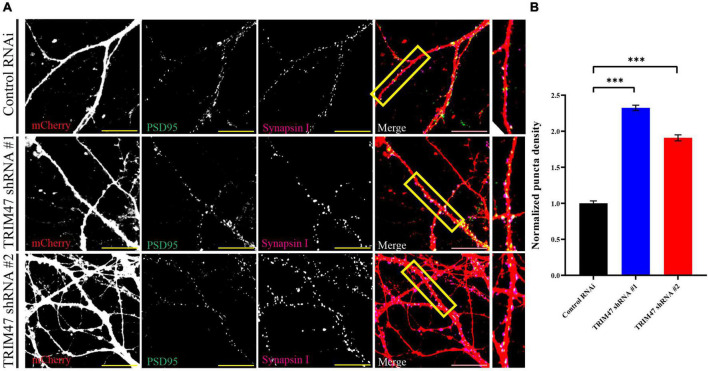
Excitatory synapse formation following TRIM47 knockdown. **(A)** Synapse density is measured from hippocampal neurons after effective knockdown of TRIM47. Neurons (DIV 3) are transduced with two distinct shRNAs against TRIM47 and these neurons (DIV 21) are immunostained using presynaptic protein Synapsin I (magenta) as well as postsynaptic protein PSD95 (green). Synapses are visualized by apposing Synapsin I and PSD95 puncta onto hippocampal neurons expressing mCherry (red). PSD95/Synapsin I colocalized puncta per micrometer of dendrite is measured to detect synapse density. Scale 20 μm. **(B)** Quantitation of synapse density is measured from neurons expressing control shRNA (Control RNAi) and two distinct shRNAs (shRNA # 1 and shRNA# 2) against TRIM47. *n* = 15–21. ^***^p < 0.001; one-way ANOVA with Dunnett’s T3 multiple comparisons test. Data represent the mean ± SEM.

Synapses are formed on dendritic spines (Amaral and Pozzo-Miller, 2009; Zito et al., 2009). Prompted by our observation that TRIM47 negatively regulates dendritic spine development, we have analyzed synapse formation in excitatory neurons of the hippocampus following loss of TRIM47 function. Hippocampal neurons (DIV 3) were transduced with lentivirus expressing two distinct shRNAs along with mCherry. These neurons (DIV 21) were immunostained with the postsynaptic protein PSD95 and presynaptic protein Synapsin I. Synapse density was measured from confocal images by analyzing the number of colocalized PSD95 and Synapsin I puncta onto mCherry expressing dendrite. We observed that the knockdown of TRIM47 significantly enhanced (0.56 ± 0.037 synapse per μm, *p* < 0.0001 for shRNA #1 and 0.46 ± 0.042 synapse per μm, *p* < 0.0001 for shRNA #2 as compared with 0.24 ± 0.032 synapse per μm for control shRNA) (Figures 6A,B) synapse density. These observations indicate that TRIM47 functions as a negative regulator of excitatory synapse development in hippocampal neurons.

## Discussion

Our study identified an E3 ubiquitin ligase that is expressed during the temporal window of synapse formation in the hippocampus. TRIM47 is localized in developing synapses (Figure 3) and its expression is regulated by neuronal activity in the developing as well as in the adult hippocampus (Figures 1, 2). The loss of TRIM47 function by two shRNAs enhances dendritic spine density as well as the number of mushroom spines without affecting dendritic complexity (Figures 4, 5). Furthermore, TRIM47 knockdown leads to an increase in glutamatergic synapse density (Figure 6). Similar phenotypic observations by distinct shRNA-mediated knockdown of TRIM47 eliminates the possibility of off-target effect of shRNA and support our observation that TRIM47 is a negative regulator of dendritic spine development and excitatory synapse formation. The localization of TRIM47 in developing synapse during the temporal window of synaptogenesis suggests that the TRIM47 could provide the spatial control of synapse formation.

Consistent with previous observation showing activity-dependent ubiquitination of synaptic proteins (Bingol and Schuman, 2006), our study suggests that the TRIM47 could function as a key regulator of activity-dependent *de novo* polyubiquitination of synaptic proteins. TRIM47 enrichment at the synapse and enhancement of its expression in hippocampal neurons by glutamate application or exposure to spatial memory paradigm indicate that TRIM47 function could spatially control spine architecture in developing as well as adult brain during learning. We observed that the NMDA receptor activation is sufficient for an increase in TRIM47 expression, and the calcium chelator EGTA blocks this enhancement. This observation suggests calcium-dependent TRIM47 expression upon NMDA receptor activation. These observations, along with previous studies, prompted us to anticipate that NMDA-mediated enhancement of TRIM47 could regulate the synaptic proteome for functional activity of the synapse (Ferreira et al., 2021).

The dendritic spine is an actin rich structure. Several factors influencing actin polymerization or depolymerization have been shown to be regulated by ubiquitin-dependent degradation by the proteasome (Schreiber et al., 2015; Lohraseb et al., 2022). We anticipate that proteasome-mediated degradation of factors influencing actin depolymerization following activity-dependent enhancement of TRIM47 could modulate the structure of the dendritic spine. The alteration of dendritic spine structure is directly correlated with the development of functional synapses and regulating synaptic potentiation during learning (Sell et al., 2021). Consistent with this logical proposition, we observed that TRIM47 also acts as a negative regulator of excitatory synapse development. It could be possible that TRIM47 promotes ubiquitination of proteins, negatively influencing synaptogenesis *via* canonical or non-canonical functions of ubiquitination.

Analyzing the impact of TRIM47 knockdown on synaptic maturation would require functional characterization of synapses by electrophysiological recording of synaptic activity and distribution of glutamatergic receptor in the synaptic compartment. To gain a mechanistic insight into the activity-regulated functions of TRIM47 in dendritic spine development and synapse formation, targets of TRIM47 needs to be identified. Detailed characterization of these targets will elucidate mechanistic aspects of TRIM47-mediated spine development and synaptogenesis.

## Data availability statement

The original contributions presented in this study are included in the article/Supplementary material, further inquiries can be directed to the corresponding author.

## Ethics statement

This animal study was reviewed and approved by the Institutional Animal Ethics Committee of National Brain Research Centre.

## Author contributions

SB conceived the idea. GS performed all experiment and analysis. SB and GS wrote the manuscript. Both authors contributed to the article and approved the submitted version.

## Supplementary material

Supplementary Figure 1 Localization of TRIM47 in rat brain. **(A)** P14 and **(B)** P60 Rat brain sections of 50 μm thickness are immunostained for TRIM47 and MAP2. Confocal images of whole brain section at ×10 (left panel), ×20 showing hippocampus (middle panel), and ×100 (right panel) shows TRIM47 localization in hippocampal neurons. Part of the image magnified is marked in yellow box.
