# Supplementary figures and images for: Activity-regulated E3 ubiquitin ligase TRIM47 modulates excitatory synapse development
Source: Front Mol Neurosci. 2022 Sep 21;15:943980. doi: 10.3389/fnmol.2022.943980 (PMC9532517; doi:10.3389/fnmol.2022.943980)

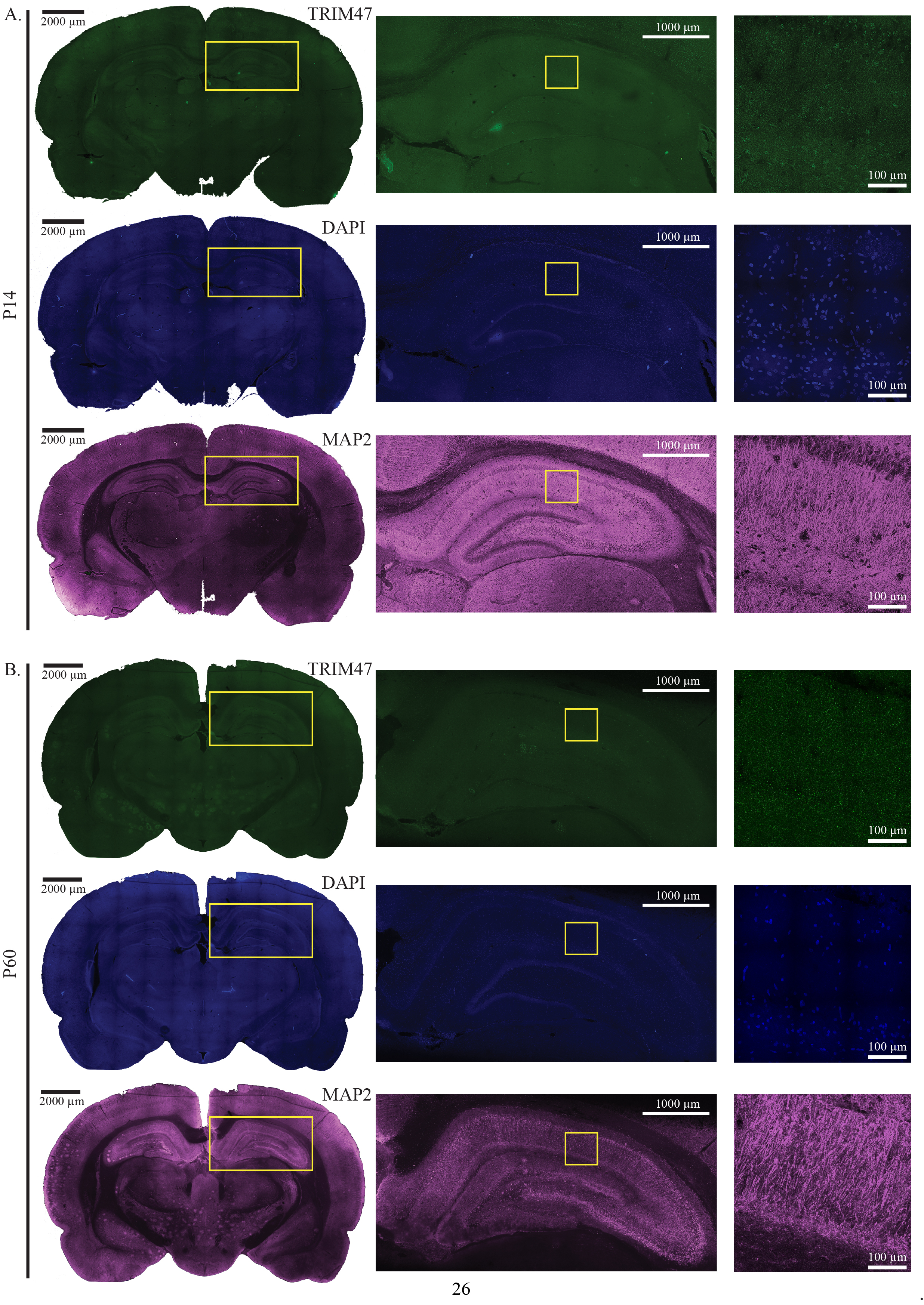

Supplement: Supplementary Figure 1 — Localization of TRIM47 in rat brain. (A) P14 and (B) P60 Rat brain sections of 50 μm thickness are immunostained for TRIM47 and MAP2. Confocal images of whole brain section at ×10 (left panel), ×20 showing hippocampus (middle panel), and ×100 (right panel) shows TRIM47 localization in hippocampal neurons. Part of the image magnified is marked in yellow box. [file Image_1.JPEG]
